# Community pharmacy professionals’ knowledge, attitudes, and practices toward substandard and falsified medicines and associated factors in Bahir Dar City, Northwest Ethiopia

**DOI:** 10.3389/fphar.2025.1523709

**Published:** 2025-02-25

**Authors:** Biset Asrade Mekonnen, Kidest Berhanu, Nebiyu Solomon, Minichil Chanie Worku, Yeniewa Kerie Anagaw

**Affiliations:** ^1^ Department of Pharmacy, College of Medicine and Health Sciences, Bahir Dar University, Bahir Dar, Ethiopia; ^2^ Department of Pharmaceutical Chemistry, School of Pharmacy, College of Medicine and Health Sciences, University of Gondar, Gondar, Ethiopia

**Keywords:** substandard, falsified, medicine, pharmacists, knowledge, attitude, practice, Ethiopia

## Abstract

**Background:**

The availability of substandard and/or falsified medicines (SFMs) in the market poses a severe threat to health and the national economy. Therefore, pharmacy professionals are highly responsible for controlling SFMs distribution in the market to improve the health of the population.

**Objective:**

The aim of this study was to assess community pharmacy professionals’ knowledge, attitudes, and practices (KAP) toward SFMs and to identify associated factors in Bahir Dar City, Northwest Ethiopia.

**Methods:**

A community-based descriptive cross-sectional study was conducted from 1 August 2024, to 30 September 2024. Participants were recruited using a simple random sampling method. A structured and self-administered questionnaire was used to collect data on sociodemographic characteristics and KAP toward SFMs. The collected data were entered and analysed using SPSS version 26. Multivariate logistic regression analysis was used to identify factors associated with participants’ KAP toward SFMs. Variables with a P value < 0.05 were considered statistically significant.

**Results:**

Of the 162 participants, 80.5% had a good knowledge and 54.9% had a positive attitude toward SFMs. However, 46.3% had a good level of practice toward SFMs. Educational levels with a master’s degree (AOR = 2.6, 95% CI: 1.06–4.35) and work experience of 21–25 years (AOR = 2.19, 95% CI: 1.79–2.80) were associated with participants’ knowledge. Educational levels with a master’s degree (AOR = 1.65, 95% CI: 0.85–2.95), work experience of 21–25 years (AOR = 1.3, 95% CI: 0.85–1.86), good knowledge (AOR: 1.21, 95% CI: 0.94–1.51), and good practice (AOR = 1.33, 95% CI: 0.85–2.01) were associated with the participants’ attitude. The practice of participants is affected by educational levels with a master’s degree (AOR = 1.2, 95% CI: 1.14–1.26), 21–25 years of work experience (AOR = 2.74, 95% CI: 1.33–5.63), good knowledge (AOR: 2.71, 95% CI: 1.50–4.92), and positive attitude (AOR = 1.06, 95% CI: 0.89–2.23).

**Conclusion:**

The study revealed that the majority of the participants had a good knowledge, and more than half had a positive attitude; however, less than half of the participants had a good level of practice toward SFMs. Education/training is required to enhance the role of community pharmacy professionals to combat their distribution and threats in the future.

## Introduction

Substandard and/or Falsified Medicines (SFMs) are medicines of poor quality, unsafe, or ineffective and cause damage because of a lack of activity or a harmful ingredient (World Health Organisation, 2018). According to the WHO, substandard medicines are authorised medicines that fail to meet either their quality standards or specifications, or both, whereas falsified medicines are medicines that are deliberately and fraudulently mislabeled with respect to their identity, composition, or source and may include products with correct or wrong ingredients, without active ingredients, with insufficient or inadequate quantities of ingredient (s) or with false packaging. They can be applied to both branded and generic products. They are often designed to appear identical to genuine medicine and, by nature, difficult to detect. They encompass several products, from life-saving medicines to lifestyle medicines; however, antibiotics and anti-malarial medicines are the most frequently reported medicines (World Health Organisation, 2017).

SFMs are a global problem; they have been reported in developed and developing countries (Shieda et al., 2012; Binagwaho et al., 2013; World Health Organisation, 2018). The World Health Organisation (WHO) estimates that around 10% of all global pharmaceutical supply is falsified and substandard, reaching up to 50% of the supply in developing countries and as low as 1% in the developed world (Glass, 2014; World Health Organisation, 2018). However, in low-and middle-income countries (LMICs), especially in sub-Saharan Africa, it is estimated that they represent 34% of the market. The internet is playing an increased role in the proliferation and consumption of SFMs (Binagwaho et al., 2013; Glass, 2014; Ozawa et al., 2018; Newton et al., 2010; World Health Organisation, 2018; Guillermo et al., 2022).

SFMs often have many public health and socioeconomic impacts in both developing and developed countries. They are a major cause of morbidity and mortality due to treatment failure, disease worsening, adverse drug reactions, drug resistance, severe economic consequences, and loss of confidence in medicine, healthcare providers, and various health systems (Johnston and Holt, 2014; World Health Organisation, 2017).

Among the total reported incidents involving health damage due to falsified medicines, 27 (56.3%) and 21 (43.7%) occurred in developing and developed countries, respectively (Mohammad et al., 2018). More than 100,000 people have died because of falsified medicines worldwide, which are thought to bring in about $75 billion annually for criminal enterprises (Esposito, 2019). Drug resistance caused by substandardisation or falsification might have contributed significantly to the inability to eradicate or control serious infections such as malaria and tuberculosis in developing countries (Akpobolokemi, et al., 2022; Cavany et al., 2023; Rasmussen et al., 2022). More than 700,000 deaths from TB and malaria have been strongly linked to SFMs worldwide (Akpobolokemi et al., 2022). According to a study conducted in Nigeria, poor-quality antimalarial drugs are responsible for 12,300 deaths and $892 million ($890-$893 million) in costs annually. If antimalarial resistance develops, the cost of malaria could increase by $839 million (11% increase, $837-$841 million), which has total economic impact of 11% and it leads 11% of total productivity loss (Beargie et al., 2019). In sub-Saharan Africa, an estimated 400,000 children are exposed to malaria and are treated with poor-quality anti-malaria medicines annually (Rasmussen et al., 2022).

**FIGURE 1:**
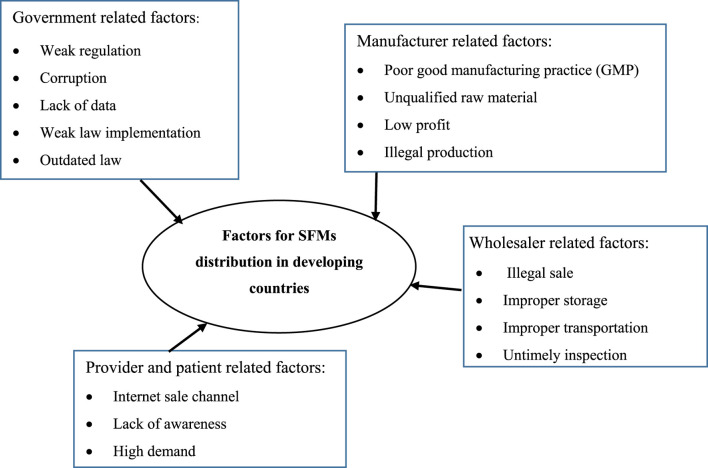
Framework of factors contributing to substandard and falsified medicine distribution.

In developed countries, SFMs are less likely to infiltrate the supply chain because of the laws on medicine regulation, production, distribution, dispensing, and consumption. However, Africa, especially sub-Saharan Africa, including Ethiopia, faces significant challenges (Newton, et al., 2010; World Health Organisation, 2018). Several factors contribute to this problem in sub-Saharan Africa, which make it easier for SFMs to infiltrate the market, and different scholars have summarised and sorted them from various perspectives (Figure 1) (Jaberidoost et al., 2013; Newton, et al., 2010; Pisani et al., 2019; Pyzik and Abubakar, 2022; World Health Organisation, 2018).

Addressing SFMs is an important global health issue, which requires collaborative efforts from healthcare professionals, regulatory authorities, and international organisations to eliminate them (Jamee et al., 2022; Pyzik and Abubakar, 2022; World Health Organisation, 2018). Tackling corruption at various levels of the pharmaceutical systems, strong regulation, updated law and increased law enforcement, establishing a pharmacovigilance system, conducting post-marketing surveillance, increasing healthcare provider and public awareness, and others are the major strategies to combat SFMs (Biwen and Zhou, 2020; Jamee et al., 2022; Jeong and Ji, 2018; Lamy and Liverani, 2015; Mensah and Toklu, 2016; Omer, 2018; Pisani, et al., 2019; Pozsgai et al., 2022; Racha et al., 2016; Sharma et al., 2021; Zhao et al., 2018).

Many studies have suggested that designing and implementing educational programs for pharmacy professionals are important to ensure the integrity of the supply chain and preventing the distribution of SFMs. Because of their expertise, accessibility, and direct interaction with patients, they are frontline healthcare professionals who can contribute significantly to safeguarding public health in both domestic and international settings. It is a unique position to educate patients about the risks associated with SFMs, the importance of obtaining medications from reputable sources, the potential dangers of online pharmacies, and the necessity of reporting any unusual effects or packaging irregularities (Wada et al., 2022; World Health Organisation, 2018; Worku et al., 2024). They should receive training to confirm the authenticity of medicines, and they can look for possible indications of falsified medicines by examining the package, labelling, and security features before patients purchase medications. Suspicious products can then be submitted to regulatory agencies for additional examination and kept off the market for patients (Chambliss et al., 2012; Jeong and Ji, 2018; Wada et al., 2022; Worku et al., 2024).

The awareness, attitudes, and practices of pharmacy professionals play a pivotal role in mitigating the distribution of SFMs in Ethiopia. However, there is a paucity of evidence based information among pharmacy professional’s awareness, attitudes, and practices toward SFMs and a cross sectional survey among pharmacy professional in Ethiopia revealed that only a tiny percentage of professionals knew the precise definition of SFMs (Eklas et al., 2021; Meshesha et al., 2022; Mishore et al., 2020; Siraj et al., 2022; Worku et al., 2024).

A cross-sectional survey conducted among healthcare providers including pharmacy professionals working in Mizan-Tepi University Teaching Hospital, Ethiopia showed that 15.8% of them described falsified medicines as product with toxic impurities and 50.3% of them were able to distinguish a falsified medicine from the genuine medicines. A very small proportion (8.2%) of the participants demonstrated that falsified medicine can be identified by physical observation of labeling, color appearance, and packaging (Siraj et al., 2022) and other additional survey conducted in Gondar, Ethiopia, revealed that pharmacy professionals had low knowledge (18.24%), attitudes (50%), and practices (35.9%) toward SFMs (Worku et al., 2024).

However, information on pharmacy professionals’ knowledge, attitudes, and practices toward SFMs in Bahir Dar has not been assessed yet. Therefore, this study aimed to assess the KAP level of community pharmacy professionals toward SFMs and to identify associated factors in Bahir Dar city, Northwest Ethiopia. The findings of this research may have significant implications for changing the KAP of community pharmacy professionals toward SFMs and reducing the incidence of SFMs. Moreover, this study will also serve as a baseline for other similar researchers to further investigate the problem.

## Materials and methods

### Study area

The study was conducted in Bahir Dar City. Bahir Dar is the capital city of the Amhara region and is located in the northwestern part of Ethiopia, with an area coverage of approximately 76.5 square kilometers (km^2^). Bahir Dar’s absolute location is within the tropics of the Northern Hemisphere, situated at 11◦270 N to 11◦430 north latitudes and 37◦140 E to 37◦380 east longitudes (Bennett and Alemie, 2016). In Bahir Dar, there are 2 comprehensive specialized hospitals, 1 governmental general hospital, 4 general private hospitals, 10 governmental health centers, 58 medium clinics, 10 specialty clinics, 10 specialty health centers, 117 community pharmacies, 93 drug stores, and 70 wholesalers.

### Study design and period

A community-based descriptive cross-sectional study was conducted from 1 August 2024, to 30 September 2024.

### Source and study population

All pharmacy professionals who worked in community pharmacies in Bahir Dar were the source of the population, whereas pharmacy professionals who worked in community pharmacies in Bahir Dar during the study period and fulfilled the inclusion criteria were the study population.

### Inclusion and exclusion criteria

Pharmacy professionals who worked in private retail outlets were included in this study, whereas allied health professionals (nurses, midwives, and physicians) were excluded.

### Sample size determination and sampling techniques

The sample size was calculated using a single population proportion formula considering a 95% confidence interval and a 5% margin of error, and the sample size was drawn as.

$$N=Z^{2}\times p\;\left(1-p\right)/W^{2}={\left(1.96\right)}^{2}\;{\left(\left(0.5*0.5\right)/0.05\right)}^{2}=384$$

Where, N = required sample size, Z = multiplier for a 95% confidence interval based on the normal distribution, p = expected prevalence, and W = desired absolute precision (5%). Therefore, the calculated sample size was 384. Because the source population in the current study was 300, which is less than 10,000, we used the following population correction formula.

$$\text{nf}=\frac{nxN}{n+N}=\frac{384x300}{384+300}=168$$

Where n = is the initial population, nf = is the corrected sample size, and N = is the total number of pharmacy professionals working in the community pharmacies in Bahir Dar. The 10% non-response rate was also added. Finally, 185 pharmacy professionals participated in the study. A simple random sampling technique was used to select participants from community pharmacies and drug stores.

#### Independent variables

Sociodemographic characteristics of community pharmacy professionals (sex, age, religion, marital status, educational level, work experience, and role).

#### Dependent variables

Knowledge, attitudes, and practices of community pharmacy professionals toward SFMs.

### Data collection tools and procedures

The data were collected by trained data collectors using self-administered closed and open-ended questionnaires, which were developed after an extensive review of related studies in the literature with a minor modification (El-dahiyat et al., 2021; Eklas et al., 2021; Kafle et al., 2024; Siraj et al., 2022; Worku et al., 2024). The questionnaire contained four sections: 1) sociodemographic characteristics of participants; 2) knowledge toward SFMs; 3) attitude toward SFMs; and 4) practice toward SFMs. A total of four pharmacists were assigned for data collection. A questionnaire was pre-tested using 5% of the participants before data collection. After a minor modification based on the feedback obtained from the pretest, 185 questionnaires were distributed to the participants.

### Knowledge assessment

There were 11 items in the knowledge section, and the participants received 1 and 0 points for correct responses and for incorrect responses, respectively. Thus, the knowledge score can range from 0 to 11. The highest possible score was 11, and the lowest possible score was 0. Those who scored greater than or equal to the mean score on these knowledge questions were considered to have “good knowledge,” and those who scored less than the mean score were considered to have “poor knowledge” toward SFMs.

### Attitude assessment

To assess the study participant’s attitude toward the SFMs, a five-point Likert scale (rating from 1 = strongly disagree to 5 = strongly agree) was used to measure the extent to which the participants agreed with the 22 items on the SFMs. The lowest and highest scores were 22 and 110, respectively. The overall attitude level was categorised using the mean score. Participants who scored greater than or equal to the mean score were considered to have “positive attitude,” and those who scored less than the mean score were considered to have “negative attitude” toward SFMs.

### Practice assessment

The participant’s practice was evaluated using 6 items, and each participant received 1 point for each correct answer and 0 points for each incorrect answer. The lowest and highest scores were 0 and 6, respectively. Participants who scored greater than or equal to the mean score were considered to have “good practice,” whereas those who scored less than the mean score were considered to have “poor practice” toward SFMs.

### Validity and reliability of the data collection tool

The reliability of the tool was determined using the Cronbach α test, and appropriate amendments were made before the main study (Cortina, 1993; Herman, 2015). This is done to ensure that the prepared questionnaires are adequately consistent and representative of the set of questions that enabled the study to answer its objectives. Eleven, twenty-two, and six items were adapted from related studies to assess knowledge (α = 0.91), attitudes (α = 0.78) and practices (α = 0.82) toward SFMs, respectively. The calculated Cronbach’s alpha value of the data collection tool was within the acceptable range (≥0.78), which demonstrated that it has an acceptable degree of internal consistency.

### Data quality assurance

Four data collectors were trained on the use of assessment tools and study objectives. Before starting the actual data collection, a pretest was done on 5% of randomly selected participants to ensure whether the study was feasible and the data collection format was appropriate and consistent in gathering the intended information on the KAP of the study participants. The pretest stage responses were not included in the final results. All collected data were double-checked for completeness, accuracy, and consistency by the investigators.

### Data analysis and presentation

The collected data were coded, entered, and analysed using SPSS version 26. The variables were coded and numbered prior to data analysis. The total KAP scores were then converted into percentages, and the KAP levels of the participants were classified based on their scores. The model’s fitness was assessed using the Homer-Lemeshow test (>0.05). Variables with a P < 0.25 in a bivariate logistic regression analysis were entered into the multivariate logistic regression analysis to identify factors associated with the participant’s KAP toward SFMs. All analyses were performed at a 95% confidence level, and variables with a p-value <0.05 in the multivariate logistic regression model were considered statistically significant factors for the KAP of the participants. The results are summarised using tables and graphs.

## Results

### Sociodemographic characteristics of the participants

Of the 185 participants, 162 were included in this study, with a response rate of 87.6%. The proportion of male participants, 91 (56.2%), was greater than that of females, 71 (43.8%), and 91 (56.2%) were aged between 21 and 30 years. Around two-thirds of the participants, 99 (61.1%) had a bachelor’s degree in pharmacy. The participants’ work experience ranged from 0 to 25 years; however, more than two-thirds, 114 (70.4%) had 0–5 years of work experience. This result revealed that most participants, 146 (90.1%), were engaged in the dispensing role (Table 1).

**TABLE 1:** Sociodemographic characteristics of the participants in Bahir Dar City, 2024 (n = 162).

Variables	Categories	Frequency	Percentage
Sex	Male	91	56.2
	Female	71	43.8
Age (in year)	21–30	91	56.2
	31–40	47	29.0
	41–50	20	12.3
	51–60	4	2.5
Religion	Orthodox	137	84.6
	Muslim	19	11.7
	Protestant	5	3.1
	Catholic	1	0.6
Marital status	Single	73	45.1
	Married	86	53.1
	Divorced	2	1.2
	Separated	1	0.6
Educational level	Diploma	57	35.2
	Bachelor’s degree	99	61.1
	Master’s degree	6	3.7
Work experience (in years)	0–5	114	70.4
	6–10	25	15.4
	11–15	17	10.5
	16–20	2	1.2
	21–25	4	2.5
Role in Pharmacy	Dispensing	146	90.1
	Pharmaceutical care	8	4.9
	Procurement	1	0.6
	Management	4	2.5
	Others	3	1.9
	Total	162	100

### Knowledge of community pharmacy professionals toward SFMs

Of the total, 143 (80.5%) participants had good knowledge toward SFMs. Of the 146 (90.1%) participants who heard about SFMs, 92 (63.0%) and 44 (30.1%) heard about falsified and substandard medicines, respectively. Although 161 (99.4%) of the study participants could easily obtain different sources of information about SFMs, 103 (63.7%) only defined SFMs correctly according to WHO guidelines, 2017. However, among all the study participants, 116 (71.6%) were aware of SFMs. Out of the total participants, 60 (37.0%) revealed that drugs with the highest risk of being SFMs were antibiotics, and 62 (38.3%) also revealed that taking these SFMs had an impact on drug resistance. Furthermore, two-thirds of the participants, 108 (66.7%), assured that pharmacists were highly involved in the SFM chain transaction, whereas 73 (45.1%) assured that the Ethiopian Food and Drug Authority is highly responsible for countering SFM transactions (Table 2).

**TABLE 2:** Pharmacy professionals’ knowledge toward SFMs in Bahir Dar City, 2024 (n = 162).

Variables	Categories	Frequency	Percentage
1. Heard about substandard and/or falsified medicines	Yes	146	90.1
	No	16	9.9
2. Types of poor-quality pharmaceutical products heard	Falsified	92	63.0
	Substandard	44	30.1
	Others	10	6.9
3. Sources of information about these products	School	40	24.7
	Colleagues	43	26.5
	Social media	23	14.2
	Health institutions	55	34.0
	I do not know	1	0.6
4. Defined correctly SFMs, according to WHO, 2017	Yes	103	63.6
	No	59	36.4
5. Awareness of substandard and/or falsified medicines	Yes	116	71.6
	No	46	28.4
6. The belief in the availability of SFMs in Ethiopia	Yes	147	90.7
	No	15	9.3
7. Institution (s) involved in the SFMs transaction chain	Hospitals	30	18.5
	Governmental clinics	18	11.1
	Non-governmental clinics	24	14.8
	Community pharmacy	32	19.8
	Importers	32	19.8
	Wholesalers	24	14.8
	I do not know	2	1.2
8. Bodies involved in SFM chain transaction	Pharmacists	108	66.7
	Physicians	25	15.4
	Paramedical personnel	19	11.7
	Drivers	7	4.3
	I do not know	3	1.9
9. Type of medicine(s) that is/are more likely to be SFMs	Antibiotics	60	37.0
	Cardiovascular drugs	3	1.9
	Herbal products	18	11.1
	Anticancer drugs	18	11.1
	Antipsychotic drugs	7	4.3
	Sexual stimulant drugs	7	4.3
	Antimalarial drugs	28	17.3
	Anti-TB drugs	7	4.3
	Food supplements	12	7.4
	I do not know	2	1.2
10. Institution(s) responsible for countering SFMs transactions	EFDA	62	38.3
	MOH	73	45.1
	EPSA	9	5.6
	Pharmacists	10	6.2
	Business owners	2	1.2
	I do not know	6	3.7
11. Impact of taking substandard and/or falsified medicines	Drug resistance	62	38.3
	Lengthening treatment duration	21	13.0
	Economic loss	33	20.4
	Death	42	25.9
	I do not know	4	2.5

Note: EFDA, Ethiopian Food and Drug Authority; MOH, Ministry of Health, Ethiopia; EPSA, Ethiopian Pharmaceutical supply agency.

### Attitudes of community pharmacy professionals toward SFMs

Of the study participants, 89 (54.9%) had a positive attitude toward SFMs. Among the study participants, 81 (50.0%) believed that SFMs were more affordable than registered original medicines, and 94 (58.1%) believed that SFMs could be very dangerous. Half of the study participants, 81 (50%) believed that the community will have a chance to buy and use SFMs, and more than half of the participants, 99 (61.1%) believed that dispensing SFMs was unethical. However, around half of the study participants, 80 (49.4%), believed that pharmacy professionals priorities profit over customer safety. The majority of participants believed that individual pharmacy professional intervention, education for both clients and pharmacy professionals, and regulatory strategies can help to prevent the dispensing of SFMs (Table 3).

**TABLE 3:** Pharmacy professionals’ attitude toward SFMs in Bahir Dar City, 2024 (n = 162).

Variables	SD (%)	D (%)	N (%)	A (%)	SA (%)
1. All pharmacy professionals are aware of SFMs	12 (7.4)	38 (23.5)	49 (30.2)	51 (31.5)	12 (7.4)
2. All non-registered medications are SFMs	17 (10.5)	11 (6.6)	13 (7.8)	101 (62.3)	21 (12.8)
3. SFMs are always poor in quality	12 (7.4)	28 (17.3)	30 (18.5)	78 (48.2)	14 (8.6)
4. SFMs are more affordable than the registered original medicines	11 (6.8)	35 (21.6)	35 (21.6)	64 (39.5)	17 (10.5)
5. It is easy to identify SFMs by their quality and price	12 (7.4)	49 (30.3)	48 (29.6)	39 (24.1)	14 (8.6)
6. SFMs can be very dangerous	11 (6.8)	30 (18.5)	27 (16.7)	67 (41.3)	27 (16.7)
7. Most SFMs are as effective as the original medicines	35 (21.6)	47 (29.0)	32 (19.8)	30 (18.5)	18 (11.1)
8. In exceptional cases, it is fine to use SFMs	35 (21.6)	59 (36.4)	34 (21.0)	32 (19.8)	2 (1.2)
9. In case of shortage of good quality medicine, it is fine to dispense SFMs to clients	39 (24.1)	67 (41.4)	24 (14.8)	25 (15.4)	7 (4.3)
10. It is fine to dispense SFMs that are not vital for disease treatment	41 (25.3)	67 (41.4)	28 (17.3)	17 (10.5)	9 (5.6)
11. In case of any harm caused by SFMs, the pharmacy professionals are the immediate responsible personnel	41 (25.3)	5 (3.1)	12 (7.4)	23 (14.2)	104 (64.2)
12. SFMs are mostly available in community pharmacies	4 (2.5)	12 (7.4)	33 (20.4)	93 (57.4)	20 (12.3)
13. Most community pharmacies knowingly dispense SFM	12 (7.4)	45 (27.8)	42 (25.9)	51 (31.5)	12 (7.4)
14. I knowingly bought SFMs in the past	22 (13.6)	43 (26.5)	47 (29.0)	43 (26.5)	7 (4.3)
15. Most likely, I have a chance to buy and use SFM	14 (8.6)	39 (24.1)	49 (30.2)	48 (29.6)	12 (7.4)
16. Most likely, my family have a chance to buy and use SFMs	11 (6.8)	38 (23.5)	55 (34.0)	44 (27.2)	14 (8.6)
17. Most likely, the community has a chance to buy and use SFMs	7 (4.3)	27 (16.7)	47 (29.0)	63 (38.9)	18 (11.1)
18. Dispensing SFMs violets professional code of ethics	6 (3.7)	32 (19.8)	25 (15.4)	56 (34.6)	43 (26.5)
19. Pharmacy professionals priorities profit over customer safety	14 (8.6)	41 (25.3)	27 (16.7)	65 (40.1)	15 (9.3)
20. The intervention of individual pharmacy professionals can prevent the dispensing of SFMs	13 (8.0)	21 (13.0)	23 (14.2)	77 (47.5)	28 (17.3)
21. Education for both clients and pharmacy professionals can help to prevent SFM dispensing	4 (2.5)	15 (9.3)	25 (15.4)	75 (46.3)	43 (26.5)
22. Regulatory strategies can help to prevent the dispensing of SFMs	6 (3.7)	15 (9.3)	14 (8.6)	68 (42.0)	59 (36.4)

Note: All statements were scored as strongly agree (SA) = 5, agree (A) = 4, neutral (N) = 3, disagree (D) = 2, and strongly disagree (SD) = 1.

### Practices of community pharmacy professionals toward SFMs

Of the total participants, 75 (46.3%) had a good level of practice in identifying SFMs. The countries of origin for these SFMs were mainly China (24.7%), followed by India (23.5%) and Ethiopia (15.4%). The participants mainly used reduced cost (39.5%) and visual inspection (28.4%) methods to identify SFMs from the original. Only 98 (60.5%) participants received training under Good Manufacturing Practice (GMP). However, more than half of the participants, 100 (61.7%), revealed that GMP training helped them to identify SFMs (Table 4).

**TABLE 4:** Pharmacy professionals’ practice toward SFMs in Bahir Dar City, 2024 (n = 162).

Variables	Categories	Frequency	Percentage
1. Have you ever experienced SFMs?	Yes	90	55.6
	No	72	44.4
2. Country of origin for the medicines you obtained	Ethiopia	25	15.4
	India	38	23.5
	China	40	24.7
	Germany	17	10.5
	Egypt	22	13.6
	UAE	14	8.6
	Others^a^	6	3.7
3. Methods used to identify SFMs	Visual inspection	46	28.4
	Reduced cost	64	39.5
	Bar code	12	7.4
	Analytical method	14	8.6
	I cannot identify	26	16.1
4. Have you taken training on medicine quality; GMP?	Yes	98	60.5
	No	64	39.5
5. If yes, does it include SFMs?	Yes	48	49.0
	No	50	51.0
6. GMP training helping you to identify SFMs	Yes	100	61.7
	No	32	19.8
	Neutral	30	18.5

^a^ Others include the USA, Japan, and Kenya.

### Factors associated with community pharmacy professionals’ knowledge level toward SFMs

Logistic regression analysis (bivariate and multivariate) was performed to assess the association between the sociodemographic characteristics of the study participants and their knowledge level toward SFMs. In the multivariable logistic regression analysis, four variables, namely, educational level, work experience, attitudes, and practices, were significantly associated with the knowledge score of community pharmacy professionals toward SFMs (P < 0.05). Those who had a master’s degree nearly three times (AOR = 2.6, 95% CI: 1.06–4.35, P < 0.001) were more likely to have good knowledge toward SFMs than those who had a diploma with controlling the other variables. Those who had 21–25 years of work experience were around twice (AOR = 2.19, 95% CI: 1.79–2.80, P < 0.001) more likely to have good knowledge toward SFMs than those who had 0–5 years of work experience. Community pharmacy professionals who had a good practice around twice (AOR = 1.77, 95% CI: 1-59-1.95, P < 0.001) more likely to have a good knowledge toward SFMs as compared to those who had a poor practice (Table 5).

**TABLE 5:** Factors associated with community pharmacy professionals’ knowledge level toward SFMs in Bahir Dar City, 2024 (n = 162).

Variables	Categories	COR (95% Cl)	AOR (95% Cl)	P-value
Sex	Male		1	
	Female	1.58 (0.64–1.74)	1.01 (0.94–1.2)	0.4
Age (in years)	21–30		1	
	31–40	1.57 (0.64–2.29)	1.54 (0.53–4.42)	0.08
	41–50	1.09 (0.94–2.32)	1.9 (0.71–4.9)	0.07
	51–60	1.67 (0.94–1.28)	2.5 (1.95–3.32)	0.06
Educational level	Diploma		1	
	Bachelor’s degree	2.57 (1.98–3.23)	1.62 (1.29–2.30)	0.02*
	Master’s degree	3.61 (2.73–4.95)	2.6 (1.06–4.35)	0.001**
Work experience (in years)	0–5		1	
	6–10	3.12 (1.85–4.4)	1.61 (1.33–9.53)	0.29
	11–15	0.96 (0.78–1.18)	1.00 (0.53–1.87)	0.04*
	16–20	2.01 (0.95–3.1)	1.15 (0.75–1.75)	0.03*
	21–25	2.81 (1.15–4.32)	2.19 (1.79–2.80)	<0.001**
Attitude	Negative		1	
	Positive	2.27 (1.54–3.35)	1.01 (0.58–1.88)	0.049*
Practice	Poor		1	
	Good	1.88 (1.6–2.4)	1.77 (1.59–1.95)	<0.001**

** Statistically significant at p < 0.001 and * statistically significant at p < 0.05.

### Factors associated with community pharmacy professionals’ attitude level toward SFMs

The educational level, work experience, knowledge, and attitudes of the community pharmacy professionals were significantly associated with their attitudes toward SFMs (P < 0.05). Community pharmacy professionals who had a bachelor’s degree (AOR = 1.41, 95% CI: 1.05–2.06, P = 0.04) and a master’s degree (AOR = 1.65, 95% CI:0.85–2.95, P = 0.03) more likely to have a good attitude toward SCMs as compared to those who had diploma. Those who had 21–25 years of work experience (AOR: 1.3, 95% CI:0.85–1.86, P = 0.03) and a good practice (AOR = 1.33, 95% CI: 0.85–2.01, P < 0.001) more likely to have a good attitude toward SFMs than those who had 0–5 years of work experiences and poor practice, respectively (Table 6).

**TABLE 6:** Factors associated with community pharmacy professionals’ attitude level toward SFMs in Bahir Dar City, 2024 (n = 162).

Variables	Categories	COR (95% Cl)	AOR (95% Cl)	P-value
Sex	Male		1	
	Female	2.08 (1.32–2.73)	0.95 (0.91–1.01)	0.08
Age (in years)	21–30		1	
	31–40	0.46 (0.53–1.18)	0.41 (0.39–2.31)	0.07
	41–50	2.12 (1.04–3.22)	2.01 (1.51–4.99)	0.25
	51–60	1.11 (0.99–1.33)	2.55 (2.0–3.5)	0.11
Educational level	Diploma		1	
	Bachelor’s degree	2.32 (1.74–2.99)	1.41 (1.05–2.06)	0.04*
	Master’s degree	2.66 (1.79–4.01)	1.65 (0.85–2.95)	0.03*
Work experience (in years)	0–5		1	
	6–10	2.1 (0.83–3.01)	1.03 (1.31–8.06)	0.1
	11–15	1.21 (1.03–1.44)	1.25 (0.79–2.12)	0.09
	16–20	1.66 (0.6–2.75)	0.82 (0.42–1.5)	0.07
	21–25	1.86 (0.2–3.4)	1.3 (0.85–1.86)	0.03*
Knowledge	Poor		1	
	Good	1.22 (0.85–2.45)	1.21 (0.94–1.51)	<0.001**
Practice	Poor		1	
	Good	1.25 (0.75–2.01)	1.33 (0.85–2.01)	<0.001**

** Statistically significant at p < 0.001 and * statistically significant at p < 0.05.

### Factors associated with community pharmacy professionals’ practices level toward SFMs

The educational level, work experience, knowledge, and attitudes of the community pharmacy professionals were significantly associated with their practices toward SFMs (P < 0.05). If the professional’s work experience increased, the level of practice also increased. Knowledgeable professionals toward SFMs were 2.7 times (AOR: 2.71, 95%CI: 1.50–4.92, P = 0.01) more likely to have a good practice toward SFMs than those who had a poor knowledge. Additionally, those who had a master’s degree (AOR = 1.2, 95%CI: 1.14–1.26, P = 0.04) and a positive attitude toward SFMs (AOR = 1.06, 95%CI: 0.89–2.23, P < 0.001) were more likely to have a good practice toward SFMs than those who had a diploma and poor attitude, respectively (Table 7).

**TABLE 7:** Factors associated with community pharmacy professionals’ practices level toward SFMs in Bahir Dar City, 2024 (n = 162).

Variables	Categories	COR (95% Cl)	AOR (95% Cl)	P-value
Sex	Male		1	
	Female	1.01 (0.85–2.53)	0.48 (0.21–1.06)	0.07
Educational level	Diploma		1	
	Bachelor’s degree	2.01 (1.05–2.79)	1.3 (0.73–2.29)	0.37
	Master’s degree	0.87 (0.65–1.08)	1.2 (1.14–1.26)	0.04*
Work experience (in years)	0–5		1	
	6–10	2.45 (1.96–3.04)	1.53 (1.05–4.83)	0.85
	11–15	1.8 (1.2–2.5)	1.3 (1.16–1.57)	0.03*
	16–20	1.66 (0.6–2.75)	2.16 (1.02–4.57)	0.045*
	21–25	3.4 (1.52–6.28)	2.74 (1.33–5.63)	0.006*
Knowledge	Poor		1	
	Good	3.01 (1.82–4.1)	2.71 (1.50–4.92)	0.01*
Attitude	Negative		1	
	Positive	1.25 (0.75–3.01)	1.06 (0.89–2.23)	<0.001**

** Statistically significant at p < 0.001 and * statistically significant at p < 0.05.

## Discussion

Controlling the worldwide substandard and falsified medicines (SFMs) distribution will prove to be a challenging task, requiring a comprehensive approach. The strategies employed by various countries differ significantly, primarily relying on their regulatory framework and the expertise of their professionals (Biwen and Zhou, 2020; Eklas et al., 2021; Jamee et al., 2022; Meshesha et al., 2022; Mishore et al., 2020; Pozsgai et al., 2022; Racha et al., 2016; Sharma et al., 2021; Siraj et al., 2022; Worku et al., 2024).

Therefore, creating awareness, a positive attitude, and good practice toward SFMs for community pharmacy professionals is one of the strategies to combat SFMs distribution and save lives. This study provides a comprehensive examination and analysis of community pharmacy professionals’ knowledge, attitudes, and practices toward SFMs in Bahir Dar city, Northwest Ethiopia.

The majority of community pharmacy professionals (80.5%) had good knowledge towards SFMs, which is consistent with a similar study conducted in Addis Ababa, Ethiopia (81.7%) (Meshesha et al., 2022). However, this result is greater than those of similar studies conducted in Gondar, Ethiopia (18.4%) (Worku et al., 2024), Alexandria, Egypt (56.0%) (Bashir et al., 2020) and Khartoum, Sudan (76.0%) (Wagiealla et al., 2020). From 90.1% of the participants who heard about SFMs, 63.0% heard about falsified medicines, and 30.1% heard about substandard medicines. They obtained information about SFMs from different sources, mainly from health institutions (34.0%), followed by colleagues (26.5%), schools (24.7%), and social media (14.2%). However, around two-thirds (63.6%) of them only knew the definition of SFMs according to World Health Organisation (2017), which is greater than other similar studies conducted in Gondar, Ethiopia (50.4%) (Worku et al., 2024), Adds Ababa, Ethiopia (37.1%) (Meshesha et al., 2022), and Alexandria, Egypt (44.0%) (Bashir et al., 2020), whereas less than a study conducted in Stockholm, Sweden (80.0%) (Persson et al., 2022).

Community pharmacy (19.8%), importers (19.8%), hospitals (18.5%), wholesalers (14.8%), non-governmental clinics (14.8%), and governmental clinics (11.1%) were involved in the SFM transaction chain in an orderly manner; however, a study conducted in Gondar reported that community pharmacy and non-governmental clinics were mainly involved in the SFM transaction chain (Worku et al., 2024).

The types of drugs more likely to be susceptible to substandardisation and falsification were antibiotics (37.0%), antimalarial drugs (17.3%), herbal products (11.1%), anticancer drugs (11.1%), food supplements (7.4%), and others (antipsychotic drugs, sexual stimulant drugs, anti-TB drugs, and cardiovascular drugs). The main institutions responsible for countering SFM transactions were EFDA (45.1%) and MOH (38.3%). Of the 97.5% of participants who knew the impacts of taking SFMs, drug resistance (38.3%), death (25.9%), economic loss (20.4%), and lengthening treatment duration (13.0%) were the major impacts, which is consistent with a similar study conducted in Gondar, Ethiopia (Worku et al., 2024).

Community pharmacy professionals who had a bachelor’s degree (AOR = 1.62, 95% CI:1.29–2.30, P = 0.02) and a master’s degree (AOR = 2.6, 95% CI: 1.06–4.35, P < 0.001) showed a significant knowledge difference toward SFMs compared with those who had a diploma. Those professionals who had 21–25 years of work experience (AOR = 2.19, 95% CI: 1.79–2.80, P < 0.001), 11–15 years (AOR = 1.00, 95% CI: 0.53–1.87, P = 0.04), and 16–20 years (AOR = 1.15, 95% CI: 0.75–1.75, P = 0.03) had a significant knowledge difference compared with those who had 0–5 years of work experience. Those who had a good practice (AOR = 1.77, 95% CI: 1.24–1.99, P < 0.001) and a positive attitude (AOR = 1.01, 95% CI: 0.58–1.88, P = 0.049) had a significant knowledge difference toward SFMs than those who had a poor practice and a negative attitude, respectively. This suggests that the educational level, work experience, attitudes, and practices affect the knowledge level of community pharmacy professionals toward SFMs.

More than half of the community pharmacy professionals (54.9%) had a positive attitude toward SFMs. This result is greater than a similar study conducted in Gondar, Ethiopia (50,0%) (Worku et al., 2024), but less than a study conducted in Khartoum, Sudan (56.0%) (Wagiealla et al., 2020). Half of the community pharmacy professionals (50.0%) believed that SFMs were more affordable than registered original medicines. More than half of the professionals (58.1%) believed that SFMs are dangerous, which was less than a similar study conducted in Kathmandu Valley, Nepal (99.71%) (Kafle et al., 2024). Less than half of the community pharmacy professionals (38.9%) believed that most community pharmacies knowingly dispense SFMs. This result is less than a similar study conducted in Lebanon (43.0%) (Hilmi et al., 2018). However, 61.1% of the community pharmacy professionals believed that dispensing SFMs is unethical, which is less than that in other similar studies conducted in Kathmandu Valley, Nepal (94.75%) (Kafle et al., 2024), Lebanon (86.5%) (Hilmi, et al., 2018), and Khartoum, Sudan (76%) (Wagiealla et al., 2020), who believed that pharmacists who dispense substandard and/or falsified medicines are unprofessional.

Of the community pharmacy professionals, 64.5% and 78.4% believed that individual pharmacy professionals’ interventions and regulatory strategies can help to prevent the dispensing of SFMs, respectively. In addition, 72.8% of community pharmacy professionals believed that education for both clients and pharmacy professionals can help to prevent SFMs dispensing. This result is consistent with other similar studies conducted in Gondar, Ethiopia (51.8%) (Worku et al., 2024) and Khartoum, Sudan (69.0%) (Wagiealla et al., 2020), who strongly agreed that education for both clients and pharmacy professionals can help to prevent the dispensing of SFMs.

The multivariate logistic regression analysis results suggest that educational level, work experience, knowledge, and practices were factors associated with the attitudes of the community pharmacy professionals toward SFMs. Those professionals who had a bachelor’s degree (AOR = 1.41, 95%CI:1.05–2.06, P = 0.04) and a master’s degree (AOR = 1.65, 95%CI: 0.85–2.95, P = 0.03) showed a significant attitude difference toward SFMs compared with those who had a diploma. Professionals who had 21–25 years of work experience had a significant attitude difference (AOR = 1.3, 95%CI: 0.85–1.86, P = 0.03) toward SFMs compared with those professionals who had 0–5 years of work experience. Knowledgeable community pharmacy professionals showed a significant difference (AOR: 1.21, 95% CI: 0.94–1.51, P = 0.001) in their attitudes compared with those who had poor knowledge, and those who had a good practice showed a significant difference (AOR = 1.33, 95% CI: 0.85–2.01, P = 0.001) in their attitudes compared with those who had a poor practice toward SFMs.

Less than half of the community pharmacy professionals (46.3%) had a good practice in identifying SFMs. However, this result is greater than other similar studies conducted in Gondar, Ethiopia (35.29%) (Worku et al., 2024) and Dubai, UAE (30.6%) (El-dahiyat et al., 2021), whereas it is lower than a similar study conducted in Yogyakarta, Indonesia (93.71%) (Kristina et al., 2023). More than half of the pharmacy professionals (55.6%) had SFMs identification experience and used different methods to identify SFMs, such as reduced cost (39.5%), visual inspection (28.4%), analytical method (8.6%), and bar code (7.4%). Of those professionals who encountered SFMs, China (24.7%), India (23.5%), Ethiopia (15.4%), Egypt (13.6%), Germany (10.5%), UAE (8.6%), and others (USA, Japan, and Kenya) (3.7%) were the countries of origin. Of the 60.5% of community pharmacy professionals who had taken GMP training, 49.0% had taken GMP training including SFMs, whereas 51.0% had taken GMP training without including SFMs. Moreover, 61.7% of the pharmacy professionals revealed that GMP training helped them to identify SFMs.

The multivariate logistic regression analysis revealed that educational level, work experience, knowledge, and their attitudes were significantly associated with their practice toward SFMs. Community pharmacy professionals who had a master’s degree (AOR = 1.2, 95% CI: 1.14–1.26, P = 0.04) showed a significant attitude difference toward SFMs compared with those who had a diploma. Those professionals who had 11–15 years (AOR = 1.3, 95% CI:1.16–1.57, P = 0.03), 16–20 years (AOR = 2.16, 95% CI:1.02–4.57, P = 0.045), and 21–25 years of work experience (AOR = 2.74, 95% CI: 1.33–5.63, P = 0.006) showed a significant difference in their practice toward SFM identification compared with those who had 0–5 years of work experience. Community pharmacy professionals who had good knowledge toward SFMs had a significant difference (AOR: 2.71, 95% CI: 1.50–4.92, P = 0.01) in their practice toward SFMs compared with those who had poor knowledge about it, and those who had a positive attitude had a significant difference (AOR = 1.06, 95% CI: 0.89–2.23, P < 0.001) in their practice toward SFMs compared with those who had a negative attitude.

### Strengths and limitations of the study

#### Strengths

This study is the first to evaluate community pharmacy professionals’ knowledge, attitudes, and practices toward substandard and falsified medicines and to identify associated factors in Bahir Dar City, Northwest Ethiopia, which could consequently determine the most appropriate strategies to combat their distribution and threats in the future. In addition, this study provides a baseline from which researchers can further investigate the problem in Ethiopia.

#### Limitations

It could not be easy to say whether the study represented the entire Amhara region and Ethiopia. The lack of previous studies in Ethiopia limits the comparison of these findings with other findings.

## Conclusion

The study revealed that the majority of community pharmacy professionals in Bahir Dar City, Ethiopia, had a good understanding. More than half had a positive attitude however, less than half had good practice toward substandard and falsified medicines. Educational level and work experience were factors associated with the knowledge, attitudes, and practices of pharmacy professionals toward substandard and falsified medicines. Increasing educational level and work experience, being knowledgeable, and having a positive attitude were significantly associated with their practice toward substandard and falsified medicines identification and prevention. Therefore, implementing regular educational programs or training through capacity building toward substandard and falsified medicines is necessary for pharmacy professionals to enhancing their role in the detection or identification of substandard and falsified medicines, strengthening medicine safety, and preventing the public from the harmful consequences of their circulation throughout the market.

## Data Availability

The original contributions presented in the study are included in the article, further inquiries can be directed to the corresponding author.

## Abbreviations

CSA, Central Statistical Agency of Ethiopia; EFDA, Ethiopian Food and Medicine Control Authority; EPSA, Ethiopian Pharmaceutical supply agency; MOH, Ministry of Health, Ethiopia; SFMs, Substandard and/or Falsified Medicines; WHO, World Health Organization.
